# Comparison of phenotypes produced in response to transient expression of genes encoded by four distinct begomoviruses in *Nicotiana benthamiana *and their correlation with the levels of developmental miRNAs

**DOI:** 10.1186/1743-422X-8-238

**Published:** 2011-05-19

**Authors:** Imran Amin, Basavaprabhu L Patil, Rob W Briddon, Shahid Mansoor, Claude M Fauquet

**Affiliations:** 1 Agricultural Biotechnology Division, National Institute for Biotechnology and Genetic Engineering (NIBGE), P O Box 577, Jhang Road, Faisalabad, ILTAB, Donald, Pakistan; 2 Danforth Plant Science Center, St. Louis, MO 63132, USA

## Abstract

**Background:**

Whitefly-transmitted geminiviruses (begomoviruses) are a major limiting factor for the production of numerous dicotyledonous crops throughout the world. Begomoviruses differ in the number of components that make up their genomes and association with satellites, and yet they cause strikingly similar phenotypes, such as leaf curling, chlorosis and stunted plant growth. MicroRNAs (miRNAs) are small endogenous RNAs that regulate plant growth and development. The study described here was aimed at investigating the effects of each virus encoded gene on the levels of developmental miRNAs to identify common trends between distinct begomoviruses.

**Results:**

All genes encoded by four distinct begomoviruses (*African cassava mosaic virus *[ACMV], *Cabbage leaf curl virus *[CbLCuV], *Tomato yellow leaf curl virus *[TYLCV] and *Cotton leaf curl virus*/Cotton leaf curl betasatellite [CLCuV/CLCuMB]) were expressed from a *Potato virus X *(PVX) vector in *Nicotiana benthamiana*. Changes in the levels of ten miRNAs in response to the virus genes were determined by northern blotting using specific miRNA probes. For the monopartite begomoviruses (TYLCV and CLCuMV) the V2 gene product was identified as the major symptom determinant while for bipartite begomoviruses (ACMV and CbLCuV) more than one gene appears to contribute to symptoms and this is reflected in changes in miRNA levels. The phenotype induced by expression of the βC1 gene of the betasatellite CLCuMB was the most distinct and consisted of leaf curling, vein swelling, thick green veins and enations and the pattern of changes in miRNA levels was the most distinct.

**Conclusions:**

Our results have identified symptom determinants encoded by begomoviruses and show that developmental abnormalities caused by transient expression of begomovirus genes correlates with altered levels of developmental miRNAs. Additionally, all begomovirus genes were shown to modulate miRNA levels, the first time this has been shown to be the case.

## Background

Plant virus infections may result in disease symptoms that can include chlorosis and/or necrosis, leaf curling, altered plant stature and morphology, presumably caused by interference with developmental processes [1]. In recent years, it has been shown experimentally that small RNAs (sRNAs), and particularly microRNAs (miRNAs), play important roles in plant development and are implicated in host-pathogen interactions [2,3]. Moreover, viroids that lack protein coding genes also cause developmental abnormalities by perturbing plant growth by interfering with small RNAs that regulate plant development [4].

miRNAs are endogenous, approx. 21nt RNAs that play important regulatory roles in animals and plants by targeting mRNA for cleavage or translational repression [5]. They are the second most abundant class of sRNAs [6] that play a very important role in multicellular organisms and influence the expression levels of many genes. Plant miRNAs have a high degree of sequence complementarity to their target mRNAs and direct the slicing of the target mRNAs in the middle of the complementary regions [7], presumably mediated by ARGONAUTE1 [8]. This has been demonstrated by the detection of 3' cleavage products that have 5' ends that start in the middle of the complementary regions. However, plant miRNAs also regulate gene expression by translational repression [9].

The geminiviruses are an important group of plant viruses with small circular, single-stranded (ss)DNA genomes that replicate in the nuclei of infected cells [10]. The incidence of diseases caused by begomoviruses has increased in recent times, presumably because of their dissemination to new hosts and enormous genetic diversity generated by recombination among these viruses. Viruses of the family *Geminiviridae *are divided into four genera based on insect vectors, host range and genome organization [11]. Whitefly-transmitted geminiviruses are classified in the genus *Begomovirus *and constitute the largest genus that causes economically-important diseases of dicotyledonous plants throughout the warmer parts of the world [12]. Begomoviruses originating from the New World are invariably bipartite, with genomes consisting of two ssDNA components, known as DNA A and DNA B, of approximately equal size (~2.8 kb). Although a few bipartite begomoviruses are known in the Old World, the vast majority are monopartite with a genome that is a homolog of the DNA A component of the bipartite viruses, and most of these interact with a group of ssDNA satellites known as betasatellites [13-15]. The major symptoms caused by begomoviruses are leaf curling, stunting and chlorosis.

The genomes of monopartite begomoviruses (and the DNA A components of begomoviruses originating from the Old World) encode six genes. The two in the virion-sense encode the coat protein (CP), involved in virus movement within and between plants, and the V2 (AV2 for the bipartite begomoviruses) protein, which is involved in virus movement in plants as well as being involved in overcoming host defences triggered by double-stranded RNA (so called RNA silencing) for some virus species [16,17]. The complementary-sense genes encode the replication associated protein (Rep; a rolling circle replication initiator protein that also interferes with host cell-cycle, the only virus encoded protein required for virus replication [18]) the replication enhancer protein (that interacts with Rep to provide a cellular environment suitable for virus replication [19] and the (A)C4 protein (that may be involved in overcoming RNA silencing and may be a pathogenicity determinant [20,21]). For bipartite, and some monopartite, begomoviruses the transcriptional activator protein (TrAP) is involved in up-regulating late, virion-sense encoded genes [22,23]. Both TrAP and the C2 protein (a TrAP homolog encoded by some monopartite begomoviruses which does not up-regulate late gene expression) may be involved in overcoming RNA silencing and up-regulating host transcription [24]. The DNA B component of bipartite begomoviruses encodes two proteins, the nuclear shuttle protein (NSP) and the movement protein (MP) which are required for inter- and intracellular virus movement [25]. All begomoviruses thus far identified in the New World are bipartite and lack the AV2 gene encoded on the DNA A component [26]. Geminiviruses encode proteins that contribute to pathogenicity. These proteins differ between monopartite and bipartite begomoviruses, as well between viruses within the individual groups. For example, the C4 protein of *Tomato leaf curl virus *(ToLCV) is a pathogenicity determinant and expression in plants results in virus-like symptoms [27]. For other monopartite begomoviruses, such as *Tomato leaf curl Java virus*, the V2 protein is the pathogenicity determinant [28] and expression of the betasatellite-encoded βC1 from a viral vector or in transgenic plants induces disease-like symptoms [29]. Many virus-encoded pathogenicity determinants have been shown to have suppressor of RNA silencing activity and to interfere in the miRNA pathway [30,31].

Viruses have acquired a variety of suppressors of RNA silencing and some even encode multiple suppressors that interfere at different steps of the silencing pathway. For geminiviruses this was first shown for the TrAP of ACMV; the protein being capable of reversing established silencing [32]. The suppression activity of the AC4 proteins from four different cassava-infecting begomoviruses were examined using the *Agrobacterium *based transient assay in *N. benthamiana *16c plants [20]. Two of the AC4 proteins, from viruses associated with recovery-type symptoms in cassava, showed suppressor activity with increased accumulation of GFP mRNA and inhibition of GFP-specific siRNAs. The AC4 proteins from non-recovery-type viruses showed little or no activity in this assay. Conversely, the TrAP proteins of the non-recovery viruses were found to be silencing suppressors, while those from recovery-type viruses were less effective in the same assay. Similarly, the V2 protein of TYLCV and the βC1 protein encoded by Tomato yellow leaf curl China betasatellite (TYLCCNB) [33,34] have suppressor activities. Expression of the ACMV AC4 protein in transgenic plants was correlated with decreased accumulation of host miRNAs and increased developmental abnormalities in *Arabidopsis thaliana *[20,35].

The study described in here was designed to further investigate the possible roles of individual genes encoded by four distinct begomoviruses in the virus infection cycle and their effects on the levels of ten miRNAs involved in plant development.

## Results

### PVX-mediated expression of *rep*

**Figure 1:**
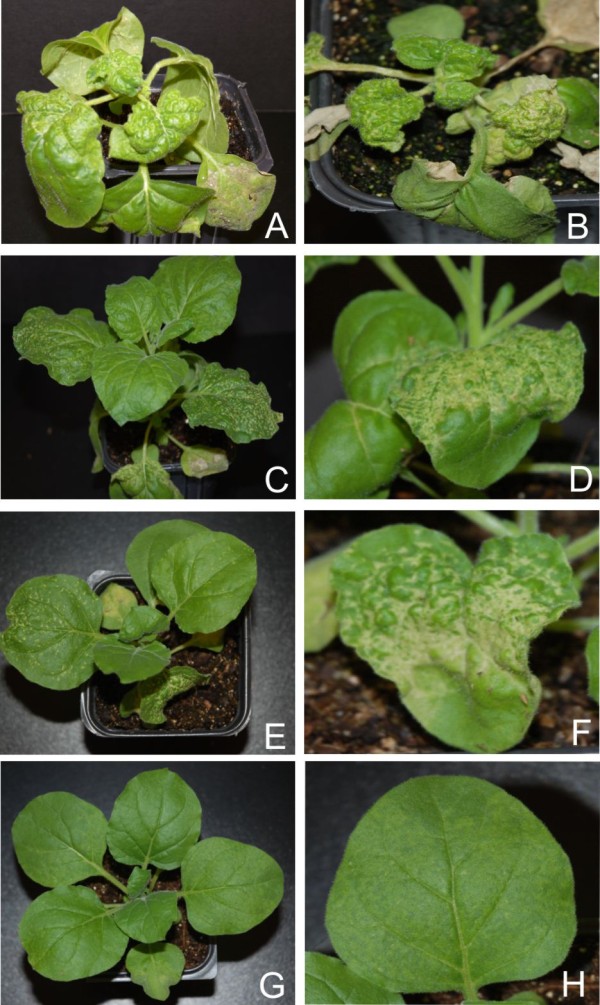
**Symptoms exhibited by *N. benthamiana *plants infected with PVX expressing the *rep *genes of selected begomoviruses**. Photographs of *N. benthamiana *plants, and close-up views of leaves, infected with PVX expressing the *rep *of ACMV (panels A and B), CLCuMV (panels C and D), CbLCuV (panels E and F). An *N. benthamiana *plant, and close-up view of a leaf, infected with the PVX vector (with no insert) are shown in panels G and H.

Inoculation of *N. benthamiana *with PVX harbouring ACMV *rep *(PVX-A-rep)resulted in a severe necrosis of the infiltrated tissues, reminiscent of a hypersensitive response (HR), that appeared approx. 6 dpi (Figure 1, panels C and D; Table 1). At approximately 8 dpi the youngest leaves, developing after inoculation, showed severe curling, vein yellowing and infected plants ceased to grow (Figure 1, panels C and D). Small necrotic lesions were also observed on the leaves developing after inoculation (Figure 1, panel B). PVX-A-rep infected *N. benthamiana *plants did not recover from the infection. In contrast, PVX infection of *N. benthamiana *resulted in mild symptoms including mild vein yellowing, very mild vein thickening and a faint mosaic that appeared approx. 10 dpi (Figure 1, panels G and H; Table 1). Additionally, at approx. 15 dpi, newly emerging leaves of *N. benthamiana *plants infected with PVX ceased to show symptoms, indicative of recovery. Infection of *N. benthamiana *with PVX harbouring *gfp *(PVX-gfp) started showing GFP fluorescence at 3 dpi which gradually increased and spread to newly emerging leaves. At 10 dpi plants also showed symptoms of PVX infection. At approx.12-14 dpi plants showed signs of recovery, with a loss of PVX symptoms and no GFP fluorescence.

**Table 1:** Summary of the symptoms induced by the expression of ACMV, CLCuMV, CbLCuV and TYLCV genes from a PVX vector in *N. benthamiana*

Gene	Construct	Latent period^□^(days)	Recovery^△^ (dpi)^▲^	**Symptoms**^#^
-	PVX	7	Yes(12-14)	MVY, VT, MMo,

*gfp*	PVX- gfp	6	Yes (12-14)	MVY, VT, Mo and GFP

	PVX-Mu-rep	7	Yes (14-16)	Nc, LCu, NcO, NcS
	
*rep*	PVX-A-rep	7	No	NcO, SLCu, VY, NcS
	
	PVX-Cb-rep	7	Yes (14-16)	NcO, MLcu, NcS
	
	PVX-T-rep	nd*	nd*	nd*

	PVX-Mu-c2	7	Yes (14-16)	NcO, MLCu, NcS
	
*trap/c2*	PVX-A-trap	5	Yes (12-14)	VY, VNc, MLCu
	
	PVX-Cb-trap	7	Yes (14-16)	VY, VNc, MLCu
	
	PVX-T-c2	7	Yes (14-16)	IVC

	PVX-Mu-ren	7	Yes (14-15)	MVY, VT, M Mo
	
*ren*	PVX-A-ren	7	Yes (14-15)	MVY, VT, M Mo
	
	PVX-Cb-ren	7	Yes (14-15)	MVY, VT, M Mo
	
	PVX-T-ren	7	Yes (14-15)	MVY, VT, M Mo

	PVX-Mu-c4	7	No	MLCu, VY, DL
	
*(a)c4*	PVX-A-ac4	7	Yes (14-16)	MVY, VT, M Mo
	
	PVX-Cb-ac4	7	Yes (14-16)	VY, LCr, VS
	
	PVX-T-c4	7	Yes (14-16)	Et, MLCu,

	PVX-Mu-cp	7	Yes (14-15)	MVY, VT, M Mo.,
	
*cp*	PVX-A-cp	7	Yes (14-15)	MLCu, Mos
	
	PVX-Cb-cp	7	Yes (14-15)	MVY, VT, MMo
	
	PVX-T-cp	7	Yes (14-15)	LCr, LCu, Mos

	PVX-Mu-v2	6	No	NcO, SLCu, VNc,
	
*(a)v2*	PVX-A-av2	8	Yes (16-18)	LCu, VY
	
	PVX-T-v2	7	No	SLCu, St, VY, NcS

*mp*	PVX-A-mp	7	Yes (14-15)	MVY, VT, M Mo,
	
	PVX-Cb-mp	7	Yes (14-15)	MVY, VT, M Mo,

*nsp*	PVX-A-nsp	7	Yes (14-15)	MLCu, NcS
	
	PVX-Cb-nsp	7	Yes (14-15)	MVY, VT, M Mo

*c5*	PVX-Ko-c5	8	Yes (14-16)	NcO, Mos,

*βc1*	PVX-Mu- βC1	6	No	SLCu, St, En, VS, VD

*Not determined.

**^△^**Newly developing leaves showing no symptoms.

**^▲^**Approx. number of days after inoculation that symptom reversion (recovery) started.

**^□^**Period between inoculation and first sign of appearance of symptoms.

^#^Symptoms are indicated as mild vein yellowing (MYV), vein thickening (VT), mild mosaic (MMo), necrosis (Nc), leaf curling (LCu), necrosis at site of inoculation (NcO), systemic necrotic lesions (NcS), severe leaf curling (SLCu), Vein yellowing (VY), mild leaf curling (MLCu), veinal necrosis (VNc), inter veinal chlorosis (IVC), deformed leaves (DL), leaf crumpling (LCr), etiolation (Et), mosaic (Mos), stunting (St), enations (En), vein swelling (VS) and vein darkening (VD).

**Figure 2:**
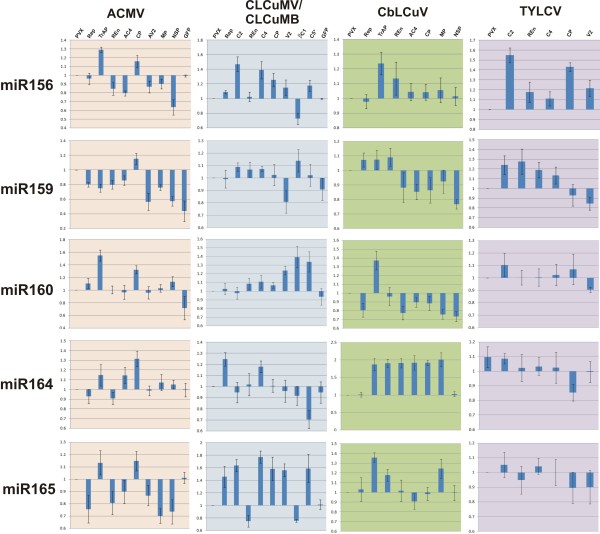
**Effects of PVX-mediated expression of begomovirus genes on the levels of miR156, 159, 160, 164 and 165**. The bar graphs show the average of two independent experiments (with standard error). The accumulation of each miRNA upon begomovirus gene expression from PVX was calculated relative to plants infected with PVX which was taken as 1. The genes used were those encoding the replication associated protein (Rep), the transcriptional activator protein (TrAP), the C2 protein (C2), the replication enhancer protein (REn), the (A)C4 protein [(A)C4], the coat protein (CP), the (A)V2 protein [(A)V2], the nuclear shuttle protein (NSP) and the movement protein (MP). Additionally the gene encoding the hypothetical protein C5 (C5*) of *Cotton leaf curl Kokhran virus *and the green fluorescence protein (GFP) were expressed from the PVX vector.

**Figure 3:**
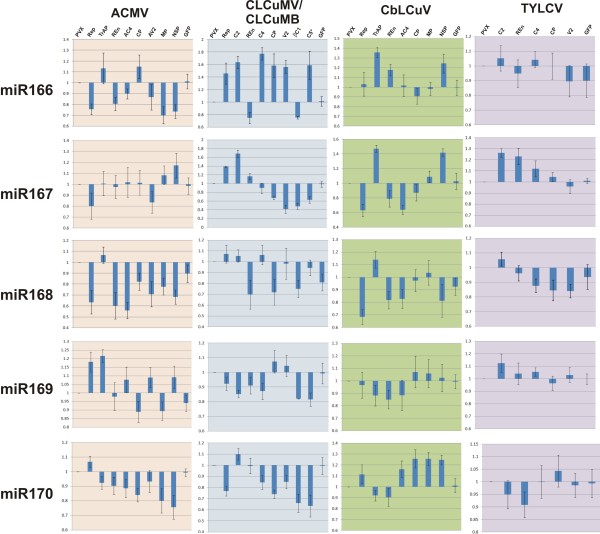
**Effects of PVX-mediated expression of begomovirus genes on the levels of miR166, 167, 168, 169 and 170**. The bar graphs show the average of two independent experiments (with standard error). The accumulation of each miRNA upon the expression of begomovirus genes from PVX was calculated relative to plants infected with PVX, which was taken as 1. The genes used were those encoding the replication associated protein (Rep), the transcriptional activator protein (TrAP), the C2 protein (C2), the replication enhancer protein (REn), the (A)C4 protein [(A)C4], the coat protein (CP), the (A)V2 protein [(A)V2], the nuclear shuttle protein (NSP) and the movement protein (MP). Additionally the gene encoding the hypothetical protein C5 (C5*) of *Cotton leaf curl Kokhran virus *and the green fluorescence protein (GFP) were expressed from the PVX vector.

Infection of PVX-A-rep in *N. benthamiana *led to a generally lower accumulation of miRNAs than evident in plants infected with PVX. A slight increase in the levels of miR156, miR159 and mir164 were observed (Figure 2 and 3). miR160 and miR170 showed an increase whereas a significant decrease in the levels of miR165, miR166, miR167 and miR168 was observed (Figure 2 and 3; Additional file 1: Figure S1).

*N. benthamiana *plants inoculated with PVX harbouring the CLCuMV *rep *(PVX-Mu-rep) developed necrosis of the infiltrated tissues at approx. 5 dpi (Figure 1, panel C). At 7 dpi leaves developing subsequent to inoculation showed a very mild downward leaf curling as well as small necrotic lesions on the veins and between the veins (Figure 1, panel D). All inoculated plants showed complete recovery, with newly developing leaves showing no symptoms, from approx. 21 dpi (data not shown). Inoculation of PVX-Mu-rep to *N. tabacum *and *N. glutinosa *induced a severe local necrosis, resembling a HR, of inoculated tissues at approximately 7 dpi. Leaves emerging subsequent to inoculation showed no signs of symptoms (data not shown). PVX-Mu-rep infection induced a pattern of miRNA accumulation which was the converse of that induced by PVX-A-rep, a general increase in the accumulation of miRNAs. There was an increase in the accumulation of miR156, miR160, miR164, miR165, miR166 and miR168, with respect to PVX infection, while a decrease in the accumulation of miR159, miR169 and miR170 was observed (Figure 2 and 3; Additional file 1: Figure S1).

PVX-mediated expression of CbLCuV *rep *(PVX-Cb-rep) in *N. benthamiana *resulted in necrosis of the inoculated tissues and virus spread systemically (Figure 1 panel F). Leaves developing subsequent to inoculation showed mild curling and necrotic lesions at approx. 8 dpi. As with PVX-Mu-rep, *N. benthamiana *plants infected with PVX-Cb-rep showed complete recovery at 21 dpi (Figure 1, panel A; Table 1). The miRNA profile of PVX-Cb-rep infected plants was generally similar to that of PVX-A-rep infections; with a general decreased level of the developmental miRNAs. A slight increase in the levels of miR160, miR165, miR166 and miR170 was detected, while a decrease in the accumulation of rest of the miRNAs was observed (Figure 2 and 3; Additional file 1: Figure S1). Unfortunately, although TYLCV *rep *could be cloned into pPGR107 and be maintained in *E. coli*, despite repeated attempts, it proved impossible to transfer the binary vector to *A. tumefaciens*. Possibly the TYLCV Rep was expressed in *Agrobacterium *and proved toxic to this bacterium species. For this reason it was not possible to assess the effects of TYLCV *rep *expression in plants.

### PVX-mediated expression of *trap/c2*

**Figure 4:**
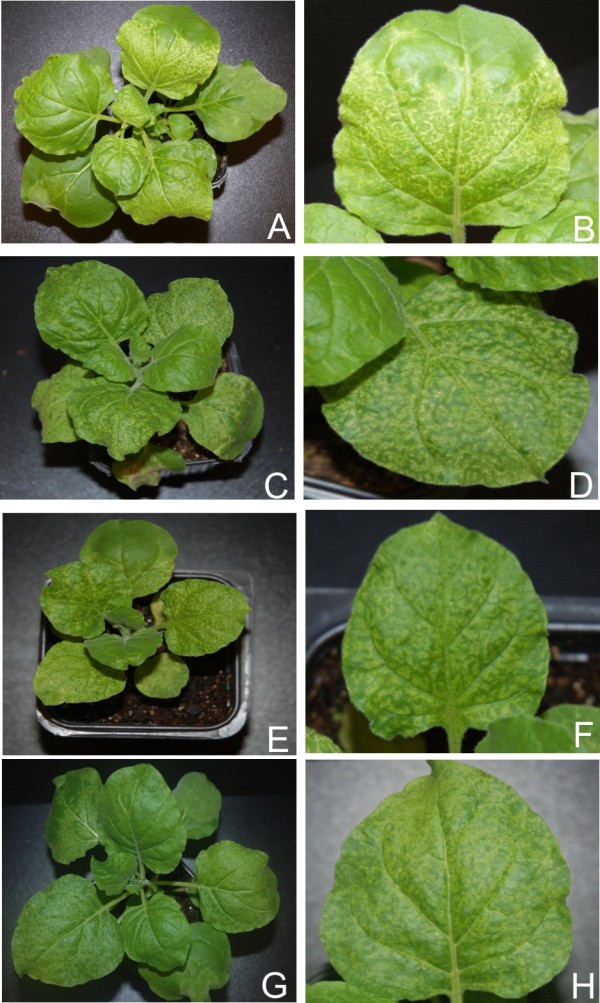
**Symptoms exhibited by *N. benthamiana *plants infected with PVX expressing the *trap/c2 *genes of selected begomoviruses**. Photographs of *N. benthamiana *plants, and close up of leaves, infected with PVX expressing the *trap/c2 *of ACMV (panels A and B), CLCuMV (panels C and D), CbLCuV (panels E and F) and TYLCV (panels G and H).

**Figure 5:**
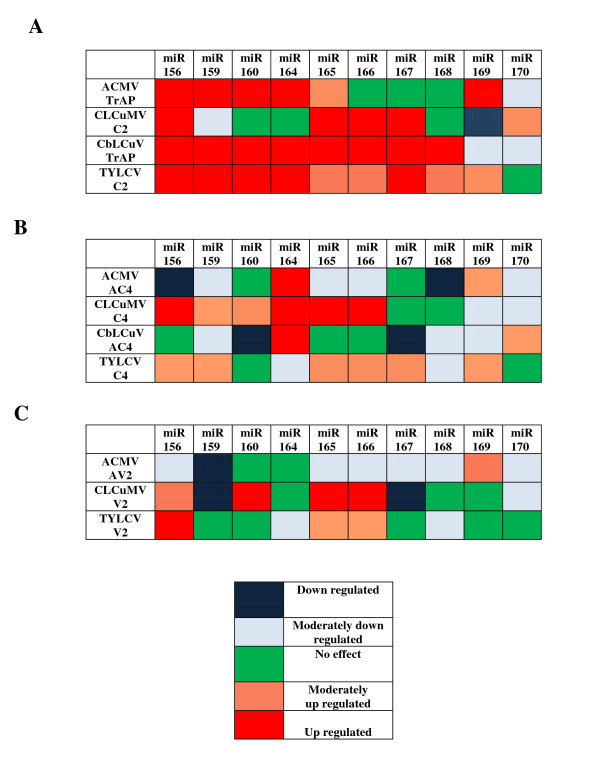
**A heat diagram summarising miRNA accumulation in response to PVX-mediated expression of selected begomovirus genes**. The results shown are for *N. benthamiana *plants infected with PVX harbouring the *trap/c2 *genes (A), *(a)c4 *genes (B) and *(a)v2 *genes (C). miRNA accumulations were compared with PVX infection alone by taking that value as 1.

Infection of *N. benthamiana *with PVX-A-trap resulted in symptoms earlier (4-5 dpi) than in plants inoculated with just PVX (7-8 dpi). Symptoms consisted of vein yellowing, veinal necrosis and mild curling of the leaves emerging after inoculation (Figure 4, panels A and B; Table 1). At approx. 15 dpi emerging leaves showed a gradual reduction in symptoms, and plants showed a complete recovery at 21 dpi (data not shown). PVX-A-trap infection in *N. benthamiana *resulted in a general increase in the levels of developmental miRNAs, with most showing a significant increase. A decrease in miR159 and miR170 was observed while the miR167 level remained unchanged in comparison with PVX infected plants (Figure 2 and 3; Additional file 1: Figure S1; Figure 5 panel A).

Inoculation of PVX harbouring the *c2 *gene of CLCuMV (PVX-Mu-c2) to *N. benthamiana *resulted in necrosis of the infiltrated tissue at 7 dpi (Figure 4 panel C; Table 1). Leaves emerging subsequent to inoculation showed mild leaf curling, vein yellowing and necrotic lesions (Figure 4, panel D). PVX-Mu-C2 infected plants showed signs of recovery at approx. 15 dpi, characterized by a gradual decrease in the symptom severity in the newly emerging leaves with later (after 21 dpi) leaves showing no symptoms, not even those typical of PVX (data not shown). PVX mediated expression of CLCuMV *c2 *resulted in an increased accumulation of developmental miRNAs in most of the cases. A significant increase was observed for the majority of miRNAs while a decrease in the levels of miR164 and miR169 was detected and the level of miR160 remained unchanged (Figure 2 and 3; Additional file 1: Figure S1).

Infection of *N. benthamiana *with PVX-Cb-trap induced symptoms at approx 7 dpi. Plants showed mild leaf curling, vein yellowing and veinal necrosis in systemic leaves (Figure 4, panels E and F; Table 1). The severity of the symptoms gradually decreased and plants showed recovery at approx. 21 dpi. The miRNA profile of PVX-Cb-trap infected plants showed a similar trend to the previously described TrAP/C2 expressing PVX constructs, consisting of a general increase in the levels of miRNA while only miR169 and miR170 showed a decrease in accumulation (Figure 2 and 3; Additional file 1: Figure S1; Figure 5 panel A).

Infections of PVX-T-c2 in *N. benthamiana *resulted in very mild symptoms. The symptoms appeared at approx. 7 dpi and consisted of mild vein yellowing, very mild vein thickening and mosaic (Figure 4, panel H; Table 1). These symptoms were slightly more severe than the symptoms produced by the PVX vector with no insert (Figure 1, panels G and H). Again recovery was evident at approx. 21 dpi. *N. benthamiana *plants infected with PVX-T-c2showed an increase in the accumulation of all miRNAs examined with the exception of miR170. Thus a general pattern of increased developmental miRNA accumulation was observed upon *trap/c2 *expression (Figure 2 and 3; Additional file 1: Figure S1; Figure 5 panel A).

### PVX-mediated expression of *ren*

**Figure 6:**
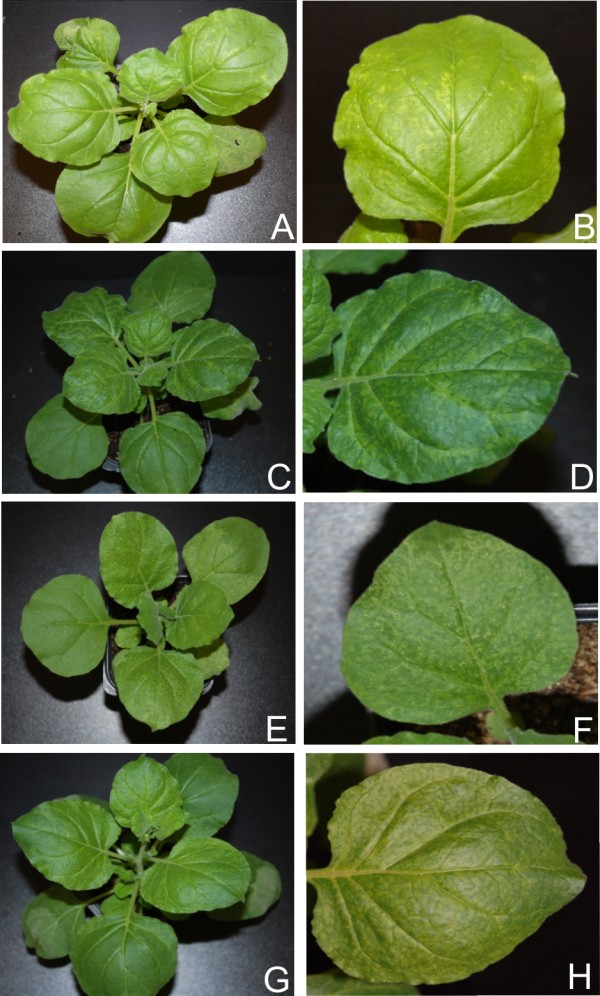
**Symptoms exhibited by *N. benthamiana *plants infected with PVX expressing the *ren *genes of selected begomoviruses**. Photographs of *N. benthamiana *plants, and close up of leaves, infected with PVX expressing *ren *of ACMV (panel A and B), CLCuMV (panel C and D), CbLCuV (panel E and F) and TYLCV (panel G and H).

For all four viruses examined, transient expression of the REn protein from PVX in *N. benthamiana *resulted in very mild symptoms that were qualitatively and quantitatively very similar. The symptoms appeared approximately 7 dpi and consisted of a mild vein yellowing, very mild vein thickening and mosaic in systemic leaves (Figure 6, panels A, B, C, D, E, F, G and H; Table 1). In all cases plants showed recovery at approx 21 dpi. PVX mediated expression of ACMV *ren *in *N. benthamiana *resulted in no significant change in the levels of miR160 and miR167 whereas there was a decrease in the accumulation of all other developmental miRNA examined. In contrast, PVX mediated expression of the *ren *genes of CLCuMV, CbLCuV and TYLCV resulted in no consistent pattern of miRNA accumulation (Figure 2 and 3; Additional file 1: Figure S1).

### PVX-mediated expression of(*a)c4*

**Figure 7:**
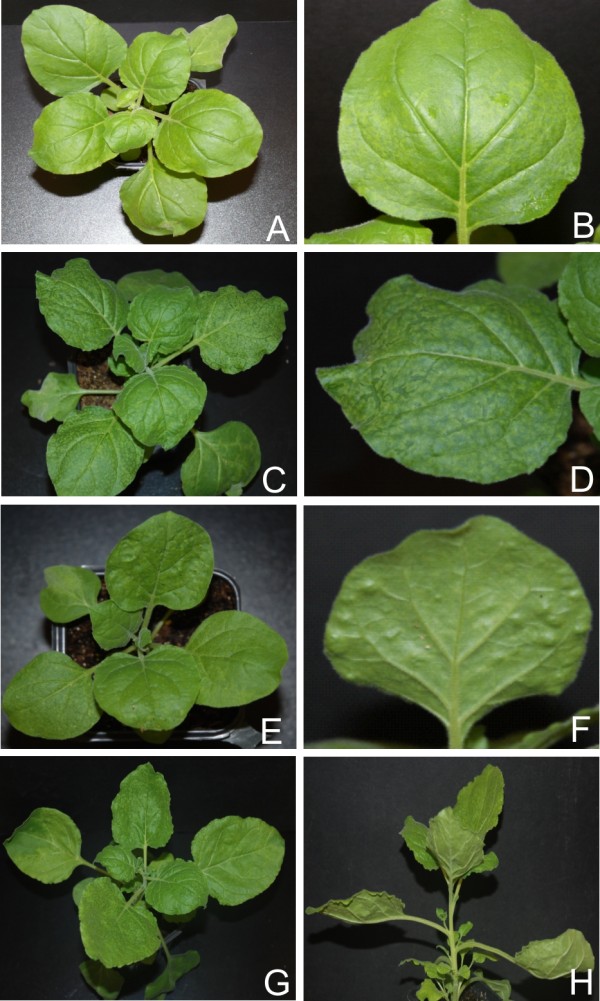
**Symptoms exhibited by *N. benthamiana *plants infected with PVX expressing the *(a)c4 *genes of selected begomoviruses**. Photographs of *N. benthamiana *plants, and close up of leaves, infected with PVX expressing *(a)c4 *of ACMV (panels A and B), CLCuMV (panels C and D), CbLCuV (panels E and F) and TYLCV (panels G and H).

PVX-mediated expression of ACMV AC4 (PVX-A-ac4) resulted in a mild phenotype which appeared at approx. 7 dpi. Symptoms consisted of a mild vein yellowing, very mild vein thickening and mosaic (Figure 7, panel B; Table 1). *N. benthamiana *plants infected with PVX-A-ac4 showed a gradual reduction in symptom severity in systemic leaves and plants eventually showed a complete recovery at approx. 21 dpi (Table 1). The miRNA profile for ACMV AC4 showed that, with the exception of miR169 (increase) and miR167 (no change), all miRNAs examined were down regulated, with the greatest decrease for miR168 (Figure 2 and 3; Additional file 1: Figure S1).

Inoculation of PVX-Mu-c4 to *N. benthamiana *resulted in symptoms approximately 7 dpi that consisted of mild leaf curling and vein yellowing. At 10 dpi emerging leaves showed a pronounced phenotype including inter-veinal yellowing and deformation of the leaves at the margins (Figure 7, panels C and D; Table 1). No recovery was observed in *N. benthamiana *inoculated with PVX-Mu-c4, with plants showing symptoms on all leaves developing subsequent to inoculation until senescence (data not shown). There was no change in the levels of miR167 and miR168 while a decrease in accumulation of miR169 and 170 was observed. An increase in the accumulation of miR156, miR159, miR160, miR164, miR165 and miR166 was observed with the greatest increase for miR165/166 (Figure 2 and 3; Additional file 1: Figure S1; Figure 5 panel B).

PVX-mediated expression of CbLCuV AC4 (PVX-Cb-ac4) resulted in a distinct phenotype. Symptoms appeared at 7 dpi and newly emerging leaves showed inter-veinal yellowing, vein swelling and leaf crumpling (Figure 7, panel E). A unique feature was the production of dimple-like lesions on systemic leaves (Figure 7, panel F). Plants inoculated with PVX-Cb-ac4 showed recovery at approx 21 dpi (Table 1). No trend in the pattern of miRNA accumulation was observed infections of *N. benthamiana *with PVX-Cb-ac4, with roughly equal numbers up-regulated, down-regulated or unaffected (Figure 2 and 3; Additional file 1: Figure S1).

Upon infection with PVX-T-c4, *N. benthamiana *showed symptoms at approx. 8 dpi that included leaf yellowing, petiole and stem elongation (showing an increase in inter-nodal distance), mild leaf curling, deformed leaf margins and leaf crumpling (Figure 7, panel H). Recovery was observed in *N. benthamiana *infected with PVX-T-c4 at approx. 21 dpi (Table 1). miRNA analyses showed that PVX mediated expression of TYLCV *c4*resulted in a general increase in the levels of developmental miRNA. No change was observed for miR160 and miR170, while a decrease was observed for miR168 (Figure 2 and 3; Additional file 1: Figure S1; Figure 5 panel B).

### PVX mediated expression of *cp*

**Figure 8:**
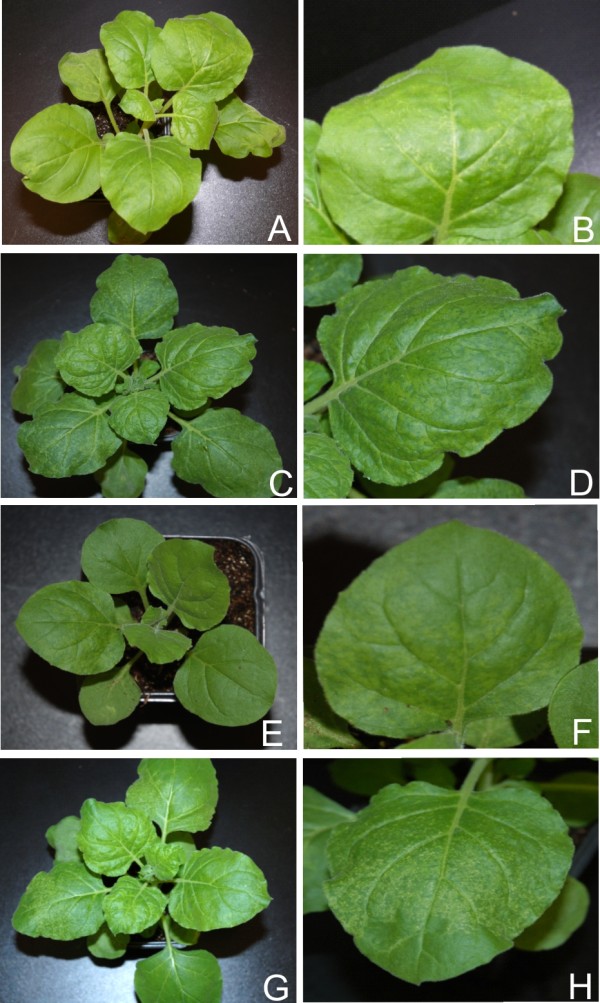
**Symptoms exhibited by *N. benthamiana *plants infected with PVX expressing the *cp *genes of selected begomoviruses**. Photographs of *N. benthamiana *plants, and close up of leaves, infected with PVX expressing *cp *of ACMV (panels A and B), CLCuMV (panel C and D), CbLCuV (panels E and F) and TYLCV (panels G and H).

Inoculation of the PVX-A-cp in *N. benthamiana *induced mild leaf crumpling approx. 10 dpi which gradually increased in severity. Emerging leaves were deformed and showed a vein yellowing phenotype (Figure 8, panels A and B). There was no effect of PVX-Mu-cp and PX-Cb-cp infection on *N. benthamiana*, with plants only exhibiting symptoms typical of PVX. A recovery in the inoculated plants was observed at approx. 21 dpi (Figure 8, panels C, D, E and F). No difference in the levels of miR167 was observed while there was a decrease in the accumulation of miR168, miR169 and miR170. A significant increase in the levels of miR156, miR159, miR160, miR164 and mir165/166 were detected (Figure 2 and 3; Additional file 1: Figure S1).

*N. benthamiana *plants infected with PVX-T-cp showed symptoms approx. 8 dpi. The symptoms included mild leaf crumpling, downward curling and mosaics that continued to increase, leaving a few green patches on the systemic leaves (Figure 8, panel H). Infected *N. benthamiana *showed recovery at approx. 21 dpi (Table 1). miRNA analyses showed that miR156, miR159, miR167 and miR170 at lower levels in comparison to the PVX control, while an increase in the levels of miR160, miR164, miR165/166, miR168 and miR169 was observed (Figure 2 and 3; Additional file 1: Figure S1).

### PVX-mediated expression of *(a)v2*

**Figure 9:**
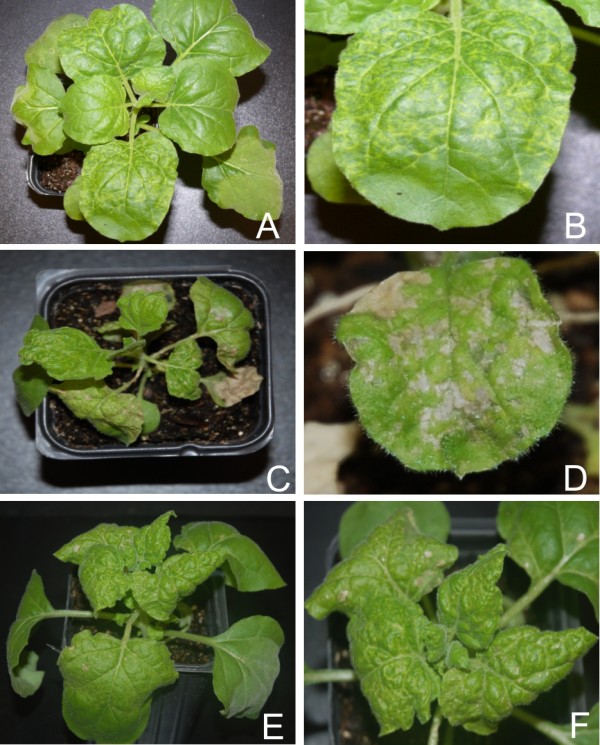
**Symptoms exhibited by *N. benthamiana *plants infected with PVX expressing the *(a)v2 *genes of selected begomoviruses**. Photographs of *N. benthamiana *plants, and close up of leaves, infected with PVX expressing *(a)v2 *of ACMV (panels A and B), CLCuMV (panels C and D) and TYLCV (panels E and F).

*N. benthamiana *plants inoculated with PVX-A-av2 showed symptoms at approx 7 dpi. Systemic leaves showed leaf curling along with vein yellowing at 8 dpi (Figure 9, panel B). Symptom severity decreased at approx. 12 dpi on the newly emerging leaves. Infected *N. benthamiana *showed complete recovery at approx. 21 dpi. With the exception of miR169, for which an increase in accumulation was found, a decrease in the accumulation of all miRNAs examined was observed (Figure 2 and 3; Additional file 1: Figure S1; Figure 5 panel C)

Infection of *N. benthamiana *with PVX-Mu-v2 induced a HR-like phenotype, characterized by cell death, at the site of inoculation at 5 dpi (Figure 9, panel C). The symptoms of infection (mild leaf curling) appeared at 7 dpi on tissues developing after inoculation. At 10 dpi there was severe downward leaf curl of systemic leaves (Figure 9, panel D; Table 1) and plants exhibited stunted growth along with necrotic lesions on the leaves which increased in size (Table 1) and eventually led to death of the plant. There was no change in the level of miR168 upon infection of PVX-Mu-v2. An increase in the levels of miR156, miR160, miR165/166 and miR169 and a decrease in the levels of mir159, miR164, mir167 and mir170 were identified (Figure 2 and 30; Additional file 1: Figure S1; Figure 5 panel C).

*N. benthamiana *plants inoculated with PVX-T-v2 showed severe symptoms. Symptoms appeared at approx. 7 dpi which increased in severity and at 11-12 dpi plants showed severe leaf curling, stunting, vein yellowing and necrotic lesions on systemic leaves (Figure 9 panel E and F). No recovery was observed in these plants even after 21 dpi (Table 1). miRNA profiling showed that PVX-T-v2 infection in *N. benthamiana *generally resulted in decreased accumulation of developmental miRNAs. With the exception of miR156, miR160 and miR170, for which a slight increase in the levels was observed, all other miRNAs under study showed an increase in the accumulation when compared with control *N. benthamiana *plants infected with PVX (Figure 2 and 3; Additional file 1: Figure S1; Figure 5 panel C).

### PVX-mediated expression of *mp *and *nsp*

**Figure 10:**
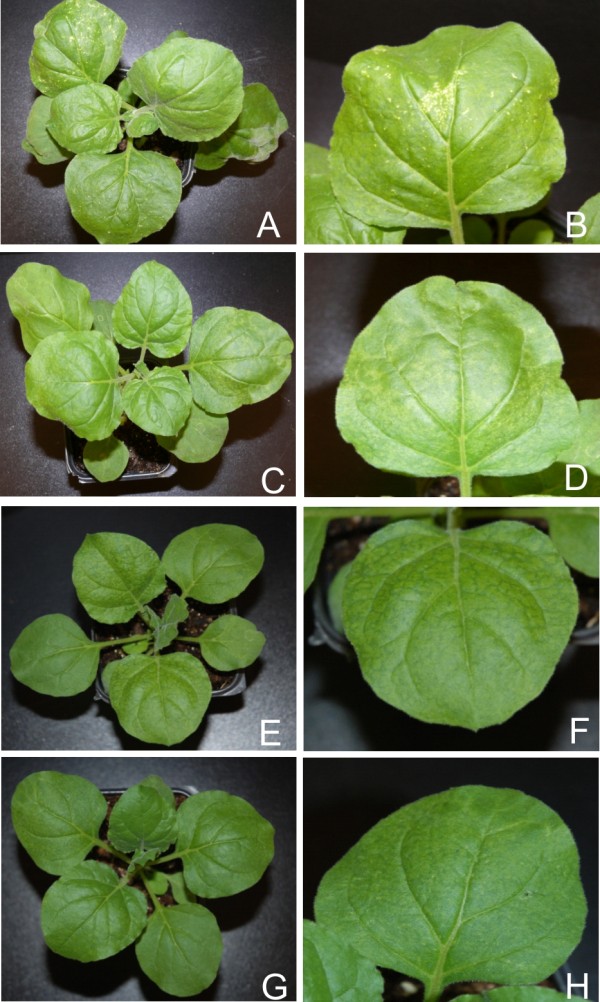
**Symptoms exhibited by *N. benthamiana *plants infected with PVX expressing the *mp or nsp *genes of selected begomoviruses**. Photographs of *N. benthamiana *plants, and close-up views of leaves, infected with PVX expressing ACMV *mp *(panels A and B), ACMV *nsp *(panels C and D), CbLCuV *mp *(panels E and F) and CbLCuV *nsp *(panels G and H).

PVX-mediated expression of ACMV *mp *(PVX-A-mp) in *N. benthamiana *induced symptoms at 8 dpi consisting of very mild leaf curling and necrosis on leaves developing after inoculation (Figure 10, panel B; Table 1). These plants showed recovery from this phenotype at approx. 21 dpi (Table 1). miRNA analyses showed that there was a slight increase in the level of miR160, miR164 and miR167 while of the remaining miRNAs examined showed a decrease in their accumulation (Figure 2 and 30; Additional file 1: Figure S1).

Infection of *N. benthamiana *with PVX-A-nsp, PVX-Cb-mp and PVX-Cb-nsp did not result in a phenotype beyond that induced by the PVX vector with no insert (Figure 10, panels C, D, E, G and H). Recovery from these phenotypes was observed in the infected *N. benthamiana *in all cases at approx. 21 dpi (Table 1). A consistent trend in the accumulation of miRNA was observed for expression of these three genes in *N. benthamiana *plants (Figure 2 and 3; Additional file 1: Figure S1).

### PVX-mediated expression of CLCuKoV *c5*

**Figure 11:**
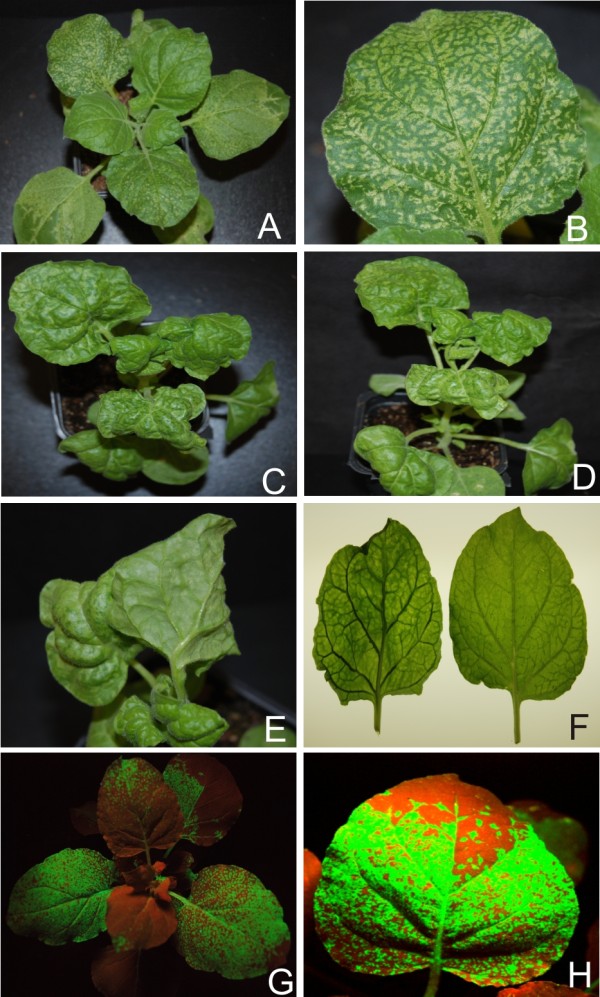
**Symptoms exhibited by *N. benthamiana *plants infected with PVX expressing the *c5 *gene of CLCuKoV, the *βc1 *gene of CLCuMB and the *gfp *gene**. Photographs of *N. benthamiana *plants, and close-up views of leaves, infected with PVX expressing CLCuKoV *c5 *(panels A and B), CLCuMB *βc1 *(panels C, D, E and F) and *gfp *(panels G and H). The photograph in panel H was taken under UV illumination to show GFP fluorescence.

Infection of *N. benthamiana *with the PVX construct expressing the *c5 *gene of Cotton leaf curl Kokhran virus (CLCuKoV; PVX-Ko-c5) showed very mild leaf curl and light green mosaics across the leaf lamina, in systemic leaves at approx. 8 dpi (Figure 11, panel A). Mosaics continued to increase in size with time and fused with each other leaving just a few dark green islands (Figure 11, panel B). Small necrotic lesions were also observed near the site of inoculation although in systemic leaves no such lesions were observed. All plants showed recovery at 21 dpi (Table 1). PVX mediated expression of CLCuKoV *c5 *in *N. benthamiana *plant showed an increase in the accumulation of miR156, miR159, miR160 and miR165/166 while there was a decrease in the accumulation of miR164, miR167, miR168, miR169 and miR170 (Figure 2 and 3; Additional file 1: Figure S1).

### PVX-mediated expression of *βc1*

PVX-mediated expression of the CLCuMB encoded *βc1 *(PVX-Mu-βc1) in *N. benthamiana *induced symptoms at approx. 7 dpi. Leaves developing subsequent to inoculation showed severe leaf curling, stunting, swollen veins and vein darkening (Figure 11, panels C and D; Table 1). A distinct feature was the formation of enations on the underside of the leaves (Figure 11, panels E and F; Table 1). Infected plants did not recover from the infection and continued to show symptoms until senescence (Table 1). miRNA profiling of *N. benthamiana *plants infected with PVX-Mu-βc1 showed a general decrease in accumulation of developmental miRNA. In most of the cases this decrease was quite significant (miR164, miR165/166, miR169 and miR170). A significant increase in the accumulation of miR159 and miR160 was also observed in infected plants (Figure 2 and 3; Additional file 1: Figure S1).

## Discussion

This is the first comparative study of the phenotypes induced by each gene encoded by CLCuMV, ACMV, CbLCuV and TYLCV and their effects on the levels of developmental miRNAs. The begomoviruses chosen for analysis represent each "subtype" of begomovirus; a true monopartite (TYLCV), a bipartite Old World (ACMV), a monopartite betasatellite-associating (CLCuMV) and a bipartite New World begomovirus (CbLCuV). The phenotypes induced were studied by expressing each gene from a PVX-based vector [36]*in planta *in the absence of the other gene products and thus may provide an insight into virus-host interaction that may not be evident by, for example, mutation of the gene in the virus. The host used in this study was *N. benthamiana*. The results have shown that homologous gene products from different viruses may induce distinct phenotypes, suggesting that these genes may interact with distinct host factors/developmental miRNAs or with similar host factors/developmental miRNA but to differing degrees. It has been demonstrated that viral suppressors of RNA silencing can interact/interfere with the miRNA pathway [30,37], although it remains unclear whether these interactions are the part of the survival strategy of viruses or just side effects of their infection cycle. Of all the genes encoded by the four viruses only three genes, *ren, mp *and *nsp *(with the exception of ACMV *mp*, that induced a noticeable phenotype), did not induce a phenotype in *N. benthamiana*, when expressed from PVX, above and beyond the mild vein yellowing symptoms induced by the vector itself.

In the study presented here ten miRNAs that play important roles in various developmental stages of the host plant were selected for analysis. It has previously been shown that PVX causes very mild symptoms in *N. benthamiana *and also causes only moderate changes in the accumulation of miR156, miR159, miR160, miR164, miR165, miR166, miR167 and miR169 [38]. Thus, PVX provides a reliable means to express genes and study their phenotypes and also their interaction with the miRNAs.

The geminivirus-encoded Rep plays a pivotal role in the viral infection cycle, probably the most important of which are its multiple functions in viral rolling circle DNA replication and interference in the host cell-cycle [18]. Generally, over expression of Rep leads to cell death [27]. This is likely due to the interaction of this protein with retinoblastoma-related protein (RBR) which is required to repress cell cycle progression. Our results have shown the PVX mediated expression of ACMV and CLCuMV Rep induce entirely different changes in miRNA expression patterns. The ACMV Rep generally decrease the miRNA levels, indicative of the increase in the level of target genes, while CLCuMV Rep results in the increased accumulation of miRNAs, suggesting a decrease in the level of target genes. No consistent pattern (either up or downward) was observed for CbLCuV Rep. A common feature of Rep of all the viruses is the decreased accumulation of miR159 which is involved in the transition of vegetative phase to sexual phase. However, this may not depict the behaviour of virus itself as it has been shown that ACMV and CbLCuV increase the accumulation of miR159 upon infection [39]. Similarly infection of ToLCNDV resulted in an increase in the levels of miR159 [40]. This particular effect caused by the Reps of begomoviruses is the opposite of that caused by REn. Down regulation of RBR expression by VIGS has been shown to induce developmental abnormalities and cell death [41]. All efforts to transform the PVX vector harbouring TYLCV *rep *into *Agrobacterium tumefaciens *were unsuccessful and this might be attributed to the toxicity of this protein. Despite this there are several reports of the production of transgenic plants expressing the Rep proteins of various begomoviruses without any apparent deleterious effects [42-44]. However, there is also a report of the inability to regenerate transgenic plants expressing the *rep *of ToLCV [27]. The production of TGMV *rep *transgenic plants which could support the replication of TGMV clones with the *rep *mutated, suggesting that fully active Rep protein was produced [42]. In contrast there are reports of production of ACMV *rep *transgenic plants which, significantly, could not support an ACMV *rep *mutant [43]. Full-length transcripts in many, but not all lines were noted. Since none of these studies report sequencing the integrated transgene, it is possible that the transformation procedure selected for transgenes did not express Rep or expressed mutated Rep, lacking the possible toxic effect of this product. Recently a single amino acid change in the Rep of ACMV has been shown to abolish the cell-death response to this protein in plants. It is thus possible that earlier results are misleading and that all geminivirus Rep proteins induce a cell-death response in plants.

REn is a protein encoded by the majority of the dicot-infecting geminiviruses that is required for optimal genome replication [19]. Surprisingly, mutation of the *ren *of TYLCV, in most cases, did not affect virus replication in tobacco protoplasts, suggesting that it is not essential for the activity of the Rep. The evidence suggests that REn acts by protein-protein interactions, since no catalytic activity has been demonstrated for this protein [19,45]. TGMV with the mutations in the *ren *gene replicated far less efficiently than their wild-type counterparts (giving lower viral DNA levels) with delayed and attenuated symptoms [46]. Expression of the ToLCV *ren *from a TMV vector caused stunting in *N. benthamiana *[27], which may be attributed to the protein interfering with cell cycle, possibly by interactions with RBR. PVX- mediated expression of TYLCV *ren *resulted in a significant increase in the accumulation of miR159. TYLCV REn interacts with at least two host-encoded proteins, PCNA and the RBR that play an important role in altering the cell cycle [19]. A major function of RBR protein is to control the expression of many genes required for cell cycle progression, by regulating the activity of E2F transcription factors [47]. The study here has shown a further way that REn may influence the cell-cycle of the host, by up-regulating miR159 that target mRNAs coding for MYB proteins which are known to bind to the promoter of the floral meristem identity gene LEAFY (LFY; [48]). The LFY gene plays an important role during the transition from the vegetative to the reproductive phase, as it is both necessary and sufficient for the initiation of individual flowers [49]. LFY is extensively expressed during the vegetative phase of plant growth [50]. Thus, the reduction in the expression of the LFY gene results in the transition from the vegetative phase to the sexual phase. In the study presented here the TrAP proteins of ACMV, CbLCuV and TYLCV induced vein yellowing and leaf curling in systemic leaves while that of CLCuMV additionally induced necrosis. The C2 of ToLCV induced necrotic lesions and some veinal necrosis on inoculated leaves of *N. benthamiana*. New leaves exhibited severe veinal necrosis extending into vascular regions [27]. The TrAP of MYMIV was found to induce HR at the inoculation site, curling of emerging leaves and necrosis and bleaching of mature leaves when expressed from a PVX vector [51]. Similarly, the C2 of *Tomato yellow leaf curl China virus *(TYLCCNV; a monopartite begomovirus) induced necrotic ringspots on inoculated leaves and necrotic vein banding on systemically infected leaves [52]. In this regard the phenotype induced by the expression of the C2 of CLCuMV is thus consistent with earlier reports. The TrAP/C2 proteins of ACMV, TYLCV and CbLCuV induced a profound phenotype which was same as that induced by that of CLCuMV, with the exception of the induction of an HR. These results suggest that TrAP/C2 proteins of these viruses interact with the host factors in a different manner as evident from the fact that in case of bipartite viruses, PVX-mediated expression of TrAPs resulted in an increased accumulation of the miR156 and for monopartite viruses C2 was associated with a decrease in the levels of miR156. This phenotype difference may be explained by the interactions of TrAP/C2 proteins with miR164. The miRNA miR164 negatively regulates several genes that encode NAC-like transcription factors [53]. These genes include CUP-SHAPED COTYLEDON 1 (CUC1) and CUC2, which are expressed in, and are necessary for, the formation of boundaries between meristems and emerging organ primordia [54]. Failure to establish organ boundaries leads to severe developmental consequences [55]. With the exception of CLCuMV, a significant increase in the levels of miR164 was observed upon PVX-mediated expression of the *trap/c2 *genes of all viruses. These results indicate that differential interaction of TrAP/C2 might be the responsible for the difference of phenotype for the CLCuMV/CLCuMB infections.

It has been shown that the TrAP/C2 proteins of ToLCNDV, *Papaya leaf curl virus *(PaLCuV) and CLCuKoV can counter a HR induced by NSP (ToLCNDV) or V2 (PaLCuV and CLCuKoV)[56,57]. Virus up-regulation of miR164 may provide a possible explanation of this phenomenon [39]. A recent study has shown that oxygen responsive elements 1 (ORE1), which is a NAC-like transcription factor, positively regulates aging-induced cell death in *Arabidopsis *leaves. ORE1 expression is up-regulated concurrently with leaf aging by ethylene insensitive 2 (EIN2) but is negatively regulated by miR164. miR164 expression gradually decreases with aging through negative regulation by EIN2, which leads to the up-regulation of ORE1 expression [58] and thus to the cell death. Up-regulation of miR164 thus will counter the cell death, and thus possibly also HR associated cell death due to NSP and V2.

The precise function of the product of the *(a)c4 *of dicot-infecting geminiviruses (with the exception of the dicot-infecting mastreviruses, which do not encode a homolog of this gene) remains unclear and may differ between viruses. Disruption of the c4 of monopartite begomoviruses results in attenuated symptoms and low infectivity, suggesting that it is involved in either symptom development, virus movement, or both [59]. The C4 of curtoviruses is an important symptom determinant but is not essential for infectivity, induces cell division [60], interacts with the brassinosteroid signalling pathway [61] and up-regulates host RKP, a ring finger protein that may interact with cell-cycle inhibitor ICK/KRP proteins, thereby interfering in the cell-cycle [62]. In contrast, early studies of the AC4 of bipartite viruses by mutagenesis concluded that his gene is either non-functional, or functionally redundant, since mutants were infectious and produced wild-type symptoms [63]. With exception of CbLCuV, PVX mediated expression of (A)C4 produced a mild phenotype, with symptoms typical of PVX. These results are consistent with earlier report for the expression of AC4 of ACMV and C4 of TYLCCNV from PVX, which resulted in no symptoms above those induced by PVX [64]. (A)C4 is highly variable among different geminiviruses. C4 of TYLCCNV was found to counter the Rep-induced HR in *N. benthamiana *[64] and the C4 of TYLCV was shown to possibly be involved in virus movement [65]. AC4 has been reported to be a suppressor of gene silencing for the bipartite begomovirus ACMV [20]. ACMV AC4 binds single-stranded miRNA and siRNA *in vitro* but does not bind the corresponding duplex forms. Thus, AC4 appears to block cytoplasmic RNA silencing, and coincidentally the miRNA pathway, by a mechanism that involves binding single-stranded siRNA and miRNA [35]. This suggests that silencing-active AC4 proteins interfere with RISC loading by acting downstream of small RNA biogenesis and duplex unwinding, possibly by facilitating the degradation of single-stranded miRNAs and siRNAs.

Expression of the *c4 *of CLCuMV and TYLCV induced mild symptoms that included leaf curling and yellowing of leaves. However, these symptoms were not as severe as reported for other viruses. For example, the *ac4 *of MYMIV expressed from the PVX vector induced virus-like symptoms (vein yellowing and leaf curl)[51]. In another report, TMV-mediated expression of ToLCV *c4 *in *N. tabacum *resulted in virus-like symptoms including rugosity, blistering and curling of leaf margins [27]. Thus the *(a)c4 *genes of CLCuMV, CbLCuV and TYLCV resemble those of ToLCV and MYMIV with respect to the phenotype induced upon expression from a virus vector. The induction of this phenotype may be attributed to a possible interference by this protein in the cell-cycle. This hypothesis is supported first by the phenotype produces by the expression of TYLCV *c4 *which resulted in etiolation (an increase in inter-nodal distance) which might be the result of cell elongation and/or cell division. BCTV upon infection of *N. benthamiana*, sugar beet and spinach induced enations. A *c4 *mutant BCTV failed to induce enations, suggesting the role of *c4 *gene in cell division (hyperplasia) and expansion (hypertrophy) of phloem parenchyma cells [66].

Our results have shown that the difference in phenotype produced by different *(a)c4 *genes may be due to differing effects on developmental miRNAs (such as miR167). It has been shown that miR167 targets auxin response factors ARF 6 and ARF8 [48]. ARF proteins regulate embryogenesis, root development and floral organ formation [67,68]. ARF6 and ARF8 regulate flower maturation [69]. Infection of plants with some begomoviruses, as well as constitutive expression of some of their genes in plants results in severe developmental defects. For example, transgenic expression of the ACMV AC4 protein in *Arabidopsis *resulted in stunted plants with severe developmental defects, such as narrow rosette leaves and lack of development of reproductive tissue [35]. Our results have shown that (A)C4 proteins of begomoviruses have differing effects on the levels of miR167 and thus that the responses of plants infected with these viruses may differ in their responses to auxin, possibly playing a part in their differing phenotypes. However, this conclusion will require further experimental verification.

The (A)C5 ORF is not well conserved between begomoviruses and is only consistently seen in the bipartite Old World begomoviruses that infect legumes (known as "legumoviruses") [51,70]. This is one of the main reasons that little attention has been paid to this potential gene. PVX-mediated expression of CLCuKoV *c5 *induced a distinct phenotype including mild leaf curling and mosaic. There are only two studies reported that have investigated the possible function of the *(a)c5 *product. In a yeast model system it was shown that this may have a role in viral DNA replication, although the study provided no indication of what that function might be [71]. In the second study, expression of the *ac5 *gene of MYMIV from the PVX vector in *N. benthamiana *induced mild leaf curl and light green mosaics on the leaves of infected plants [51], symptoms very similar to those obtained for CLCuKoV *c5 *here. Although *ac5 *is consistently present in legumoviruses, it is interesting to note that no study has mapped the transcripts of MYMIV. A study of the closely related MYMV did not identify transcripts spanning the *ac5 *ORF [72]. However, this was not an exhaustive study, aiming to identify only the major transcripts, and it is thus possible that minor transcripts suitable for translation of the AC5 were not characterized. The phenotype induced by the expression of CLCuKoV *c5 *indicates that the product of the gene has an effect on cellular metabolism but gives no indication of what the basis for that effect might be. Nevertheless, the identification of an effect as well as the fact that *c5 *appear to reduce the PVX latent period and delay the onset of recovery (Table 1) indicates that further studies are warranted to investigate the potential contribution this gene product has for the viral infection cycle. The effect of mutagenesis of this gene on virus infectivity now needs to be investigated to determine whether it has any function in the virus infection cycle and transcript mapping to determine whether suitable mRNAs are produced from which it could be translated, which would rule out it possibly being an evolutionarily conserved pseudo-gene. Our results have shown that upon PVX mediated expression of *c5 *there is a generally decrease in the accumulation of miRNAs. This decrease in the accumulation in miRNAs is quite significant. Thus there is a significant increase in the expression of target genes that lead to a severe phenotype produced by CLCuKoV *c5 *gene. This may also indicate that the C5 protein results in a general up-regulation of the target genes in the cell.

There are only two published reports on transient expression of the CP of geminiviruses. The first is the expression of the CP of ToLCV from a TMV based vector in *Nicotiana *spp. which induced no discernable phenotype [27]. The second is a recent report of PVX-mediated expression of the MYMIV CP which induced leaf crumpling and vein yellowing in *N. benthamiana *[51]. However, earlier studies of ACMV and TYLCV that produced of transgenic *N. benthamiana *and tomato lines, respectively, constitutively expressing the CP encoding genes do not refer to any phenotype [73]. The lack of symptoms induced by the PVX mediated expression of CP of CLCuMV and CbLCuV are thus in agreement with these earlier studies. However, transient expression of ACMV and TYLCV CP induced leaf crumpling and vein yellowing in *N. benthamiana*, is thus somewhat unusual but still in agreement with earlier reports where it was shown that PVX-mediated expression of MYMIV induced leaf deformation and showed a pronounced vein yellowing in *N. benthamiana *[51].

The CP is the only structural protein of geminiviruses; it forms the typical geminate particles from which the family derives its name. It is vector determining and thus has to interact with both plant hosts and arthropod vectors [74]; the CP of TYLCSV has recently been shown to interact with a *B. tabaci*-encoded heat shock family protein [75]. It plays a part in virus movement and for monopartite begomoviruses it is essential, mutation of the gene abolishes infectivity [76]. Although it is not essential for the movement of bipartite begomoviruses, viruses with mutations in the CP gene show extended latent periods, indicating that even for these viruses CP plays a part in movement [63]. The CP has sequence non-specific DNA binding properties [77,78] and it is likely that CP-ssDNA interactions are required for particle formation (since no empty, thus lacking DNA, virus particles have been reported). Studies with TYLCV have shown that the CP acts as a functional homolog of the bipartite begomovirus NSP, having both nuclear localization signals (NLS) and nuclear export signals [79] and interacts *in planta *with a karyopherin α that likely is involved in transport into the nucleus [80]. In another relevant study, using GFP tagging and *N. plumbaginifolia *protoplast, it was shown that the CP of MYMV contains two NLSs and interacts with karyopherin α [81]. However, none of these earlier studies provides any concrete indication as to why the CPs of ACMV and TYLCV appear to affect plant development or why the CP of these viruses are different from all others so far examined, highlighting instead the inconsistency between the transgenically expressed situation (no apparent symptoms) and the PVX expressed situation (a mild symptom phenotype). A possible explanation is that CP is a functional homolog of NSP of bipartite viruses which, for *Tomato leaf curl New Delhi virus *(ToLCNDV), has been shown to be a pathogenicity determinant [82]. Thus possibly the CPs of ACMV and TYLCV have some of the same interactions, involved in shuttling DNA into/out of the nucleus as NSP. However, this would not explain the differences between ACMV/TYLCV and CbLCuV/CLCuMV, unless the interactions are host specific. A far more likely explanation is that the symptoms are due to tissue specific expression. PVX, in common with many phytopathogenic viruses, is transported to the sink leaves via the phloem and is there unloaded (in class I-III veins) [83]. These studies also showed that only a limited number of cells are infected, mostly immediately adjacent to the phloem, and therefore a gene expressed from a PVX-vector will likely only be expressed in a limited number of cells close to the phloem. In contrast, transgenic expression of genes driven by the Cauliflower mosaic virus 35S promoter should (at least in theory) lead to expression in every cell. Thus this difference in expression pattern may be the reason why transgenic and PVX-mediated expression of (at least some) begomoviruses CPs contrast with respect to induction of disease-like symptoms. Clearly the way to answer this question will be to produce transgenic plants expressing the CPs under a tissue-specific (phloem-specific) promoter to see whether this reproduces the symptoms seen in plants when expressing the CPs from PVX.

Infection of *N. benthamiana *with PVX-A-AV2 resulted in vein yellowing and leaf curling. These results are in agreement with the PVX-mediated expression of AV2 of EACMCV that similarly induced a mild mottling [84]. V2 of TYLCV, when expressed from PVX in *N. benthamiana*, induced symptoms consisting of yellowing, leaf curling and stunting. The results presented here are also in line with earlier reported studies where it was shown that expression of the V2 of ToLCV, from a TMV vector, caused stunting of and yellowing in *N. benthamiana*, symptoms reminiscent of infections involving this virus [27]. The symptoms induced by expression of the TYLCV V2 from the PVX vector closely mimic the symptoms of TYLCV in *N. benthamiana*. This suggests that the V2 is possibly the major symptom determinant of both TYLCV and ToLCV. It was shown that PVX-mediated expression of the *βc1 *of CLCuMB induced all the symptoms in *N. benthamiana *that are typically induced in this host by CLCuMV together with CLCuMB [29]. In common with PVX [83], TYLCV is phloem limited [85]. This thus suggests that TYLCV V2 can induce all the symptoms typical of tomato yellow leaf curl disease when expressed in the correct tissues. A similar conclusion was reached for the betasatellite-encoded *βc1 *of CLCuMB[29]. This gene was shown to induce all symptoms typical of CLCuD, when expressed from the PVX vector, including the characteristic enations. PVX mediated expression of CLCuMV V2 resulted in severe leaf curling followed by a HR in *N. benthamiana*. This indicates that the cell death was unable to contain PVX. This HR like phenomena upon inoculation of V2 of CLCuMV indicates that this gene might be a pathogenicity determinant and a target of host defense responses. A possible reason of these virus-like symptoms could be an altered level of hormones. This hypothesis is supported by our finding that PVX mediated expression of CLCuMV V2 resulted in a marked increase in the level of miR160 which is responsible for interaction with ARFs.

All functions thus far ascribed to betasatellites have been shown to be mediated by the single, complementary-sense gene-product, βC1, they encode. βC1 is a pathogenicity determinant [86], a suppressor of PTGS [16,34], binds DNA without size or sequence specificity [34] and may be involved in virus movement [87]. Constitutive expression of βC1 in transgenic plants, in the absence of helper virus, induces "virus-like" symptoms but these do not resemble the typical symptoms of the intact betasatellite in association with its helper begomovirus [88]. For example, expression of the *βc1 *gene of *Ageratum *yellow vein betasatellite (AYVB) yielded *N. benthamiana *plants with severe developmental abnormalities (severely twisted stems and petioles) and vein greening [89], whereas infection of AYVB in the presence of its cognate helper begomovirus, AYVV, results in plants with a severe downward leaf curling phenotype. However, PVX-mediated expression of CLCuMB βC1 induced symptoms in *N. tabacum *reminiscent of the symptoms cause by CLCuMV with CLCuMB including the formation of enations [29]. Thus the results presented here for PVX mediated expression of CLCuMB βC1 in *N. benthamiana *are consistent with earlier reports. The symptoms induced consisted of leaf curling, vein yellowing, stunting and enations; symptoms that are typical of cotton leaf curl disease in cotton [90] and *N. tabacum *[13]. This indicates that, although the βC1 is the major symptom determinant of the CLCuMV-CLCuMB complex, the *bona fide *disease symptoms are only produced when this is expressed in the correct tissues, with the implication that PVX and CLCuMV have similar, phloem limited, tissue specificities. Our finding have shown that PVX mediated expression of βC1 of CLCuMB resulted in a significant change in miRNA levels, generally a decrease in miRNA accumulation which indicate that infection of βC1 usually increase the expression of host genes as evident from the decrease levels of miRNAs which in turn increase the level of target gene. However, this study is based on ten miRNAs. A more detailed study is required to confirm this hypothesis.

The host responses observed in the present study result from high level and unregulated expression of individual genes from a PVX based vector. The phenotypes induced are thus likely to be exaggerated. In actual virus infections transcription of all viral genes is tightly regulated, both spatially and temporally. For example, Rep is an early gene required for genome replication that represses its own expression at the level of transcription [91], probably to avoid the toxic effects of the protein were it to accumulate to higher than required levels. The results presented here suggested that all genes encoded by begomoviruses interfere with host processes during viral infection by interacting with, or at least affecting the levels of, miRNAs. Some unusual symptom phenotypes were observed. Although CbLCuV has not previously been associated with the induction of hypo- and/or hyperplasia, PVX-mediated expression of AC4 nevertheless was shown to induce vein swelling, likely due to hypo-/hyperplasia, pinpointing this genes as a major contributor to the leaf curl symptoms seen in CbLCV infections. Similarly, TYLCV V2 expression induced virus-like symptoms typical of TYLCV infections of *N. benthamiana*, highlighting this gene as the major pathogenicity/symptom determinant. Thus although in some cases no meaningful conclusions could be drawn, the results obtained have highlighted some interesting effects that warrant further investigation. In particular the study demonstrated that some proteins from distinct begomoviruses induce differing effects on host miRNAs, such as the CP. Another point to keep in mind is that the four viruses examined have distinct host adaptations. CLCuMV/CLCuMB is adapted to hosts in the *Malvaceae *(specifically cotton), TYLCV to hosts in the *Solanaceae *(specifically tomato), ACMV to *Euphorbiaceae *(specifically cassava) and CbLCV to plants of the *Brassicaceae *(specifically cabbage), and the differences may thus result from expression in a heterologous host (*N. benthamiana*). It is therefore important, in the future, to repeat these studies in the natural host to verify the results. This should be possible in tomato and cabbage, for which suitable expression vectors are available. However, at this time no suitable vectors are available for either cotton or cassava and it is thus not possible to repeat these studies in the natural host at this time. Our findings have shown that even if the symptoms produced by begomovirus gene products are similar, the effects on the levels of miRNAs may differ. As well as the begomovirus-encoded proteins previously shown to have suppressor activity and, at least in some cases, to bind small RNAs [(A)V2, TrAP/C2 and (A)C4)] the analysis has shown that the other (non-suppressor) proteins can affect miRNA levels, the first time this has been shown to be the case. Nevertheless, at least four of these proteins (MP, NSP, Rep and CP) have nucleic acid binding activities, of which only one (Rep) is sequence-specific. At the moment, there is no evidence to suggest that they can directly bind small RNAs such as miRNAs. Since these apparently do not have a direct effect on miRNAs, by binding them so as to remove them from the system and thereby up-regulate the genes they control, the implication is that their effects on miRNA levels is indirect, by affecting host factors that in turn affect miRNA levels. Consistent with the idea it has recently been shown that *Oilseed rape mosaic virus *infection up-regulates transcription from the miR164a promoter [92]. Only one begomovirus-encoded protein, TrAP, acts as a transcription factor. For many begomoviruses TrAP acts to up regulate the promoter of the late, virion-sense virus-encoded genes and also up or down regulates numerous host genes. Additionally the TrAP of MYMV has been shown to up regulate 162 *Arabidopsis *genes in a microarray analysis [24] and all begomovirus-encoded proteins have been shown to interact with one or more host proteins. There is thus a multitude of possible ways for begomoviruses to affect miRNA levels and results presented here suggest that all begomovirus-encoded proteins may play a part in this.

## Methods

### Production of expression constructs

All the genes encoded by begomovirus isolates CLCuMV-His[PK:Mul] (AJ496461), ACMV-[CM:DO3:98] (DNA-A, AY211885; DNA-B, AF112353), CbLCuV-[US:Flo:96] (DNA-A, U65529; DNA-B, U65530) and TYLCV-Mld[ES:72:97] (AF071228) were amplified with specific primers (Additional file 2: Table S1) and cloned into pTZ57R/T using an InsTAclone PCR cloning kit (Fermentas). The genes were then transferred to pGR107 [36] using restriction endonucleases *Cla*I, *Sma*I or *Sal*I sites that were introduced, in the primers used for amplification. A *gfp V *gene was also cloned in pGR107. The primers for the *gfp *gene were designed on sequenced reported by [93].

### Agroinoculation

pGR107 and constructs were electroporated into *Agrobacterium *strain GV3101. For agroinoculation, glycerol stocks of *Agrobacterium *harbouring gene constructs were streaked on solid AB minimal medium plates containing 12.5 μg/ml rifampicin and 50 μg/ml kanamycin and incubated at 28°C for 48 hours. A single bacterial colony was picked with a sterile wire loop and inoculated into 50 ml LB medium containing the required antibiotics and placed in a shaker (160 rotations per minute) at 28°C until the OD600 of the culture was 1. The cells were harvested by centrifugation at 3220 ×g for 8 minutes and resuspended in LB medium containing acetosyringone (final concentration 100 μM). For agroinoculation to *N. benthamiana*, plants at the 4 to 5 leaf stage were selected and were not watered for 24 hours before inoculation. Young leaves were infiltrated by gently appressing a 5 ml syringe to the abaxial surface and depressing the plunger until a water-soaked appearance was achieved.

### Plant growth conditions and sample collection

*N. benthamiana *were grown at 28°C with 16 hours dark period/8 hours light period and 65% humidity in small 5 inch diameter plastic pots containing clay, silt, sand and compost in equal proportions. All plants were watered on daily basis and with Hoagland solution (0.75 mM MgSO4.7H2O, 1.5 mM Ca(NO3)2.4H2O, 0.5 mM KH2PO4, 1.25 mM KNO3, micronutrients [50 μM H3BO3, 15 μM MnCl2.4H2O, 2.0 μM ZnSO4.7H2O, 0.5 μM Na2MnO4.2H2O, 1.5 μM CuSO4.5H2O] and Fe-EDTA [30 μM FeSO4.7H2O, 1 mM KOH, 30 μM EDTA.2Na] once a week if required. Samples were collected 12 days post inoculation (dpi) and photographed with a high resolution camera. Systemic young leaves of inoculated plants were collected, kept in plastic bags labelled with permanent marker, transported on ice and stored at -80°C until utilized.

### RNA extraction and miRNA analysis

Total RNA was extracted from infected plant material and northern blot analyses were conducted as described previously [39]. Oligonucleotide primers complementary to *Arabidopsis *miRNAs were end-labelled, as described earlier [39], using a DIG oligo labelling kit according to manufacturer instructions (Roche). Membranes were washed three times using 2× SSC, 0.1% [w/v] SDS and exposed to X-ray film after treatment with CDP-Star (Roche, Switzerland), according to the manufacturer's instructions.

The intensity of each band on X-ray films was quantified using ImagJ software. Data from these analyses were used to normalize the intensity of each band, based on the amount of rRNA loaded in each well. For PVX mediated viral gene expressions, the value for the miRNA species in PVX alone infected *N. benthamiana *plants were set at 1 and other data was calculated relative to this value. The data shown is the averages of two independent biological replicates along with standard deviations (SD).

## Competing interests

The authors declare that they have no competing interests.

## Authors' contributions

IA, BP performed the experiments. IA, SM, RWB and CMF were involved in data analysis and preparation of the manuscript. All authors read and approved the final manuscript.

## Supplementary Material

Additional file 1 **Figure S1: Effects of PVX-mediated expression of begomovirus genes in *N benthamiana *on the levels of selected miRNAs**. Northern blot analysis to detect the accumulation of selected miRNAs after infection with PVX and PVX expressing begomovirus-encoded genes. The genes used were those encoding the replication associated protein (Rep), the transcriptional activator protein (TrAP), the C2 protein (C2), the replication enhancer protein (REn), the (A)C4 protein [(A)C4], the coat protein (CP), the (A)V2 protein [(A)V2], the nuclear shuttle protein (NSP) and the movement protein (MP) Additionally the gene encoding the hypothetical protein C5 (C5*) of *Cotton leaf curl Kokhran virus *and the green fluorescence protein (GFP) were expressed from the PVX. Shown below the northern blots in each case are the rRNA bands of the ethidium bromide-stained agarose gels that were used to normalize the data for loading. click here for file

Additional file 2 **Table S1: Primers used to PCR amplify begomovirus genes and *gfp*for cloning in the PVX vector**. click here for file
