# Supplementary figures and images for: Comparison of phenotypes produced in response to transient expression of genes encoded by four distinct begomoviruses in Nicotiana benthamiana and their correlation with the levels of developmental miRNAs
Source: Virol J. 2011 May 19;8:238. doi: 10.1186/1743-422X-8-238 (PMC3166278; doi:10.1186/1743-422X-8-238)

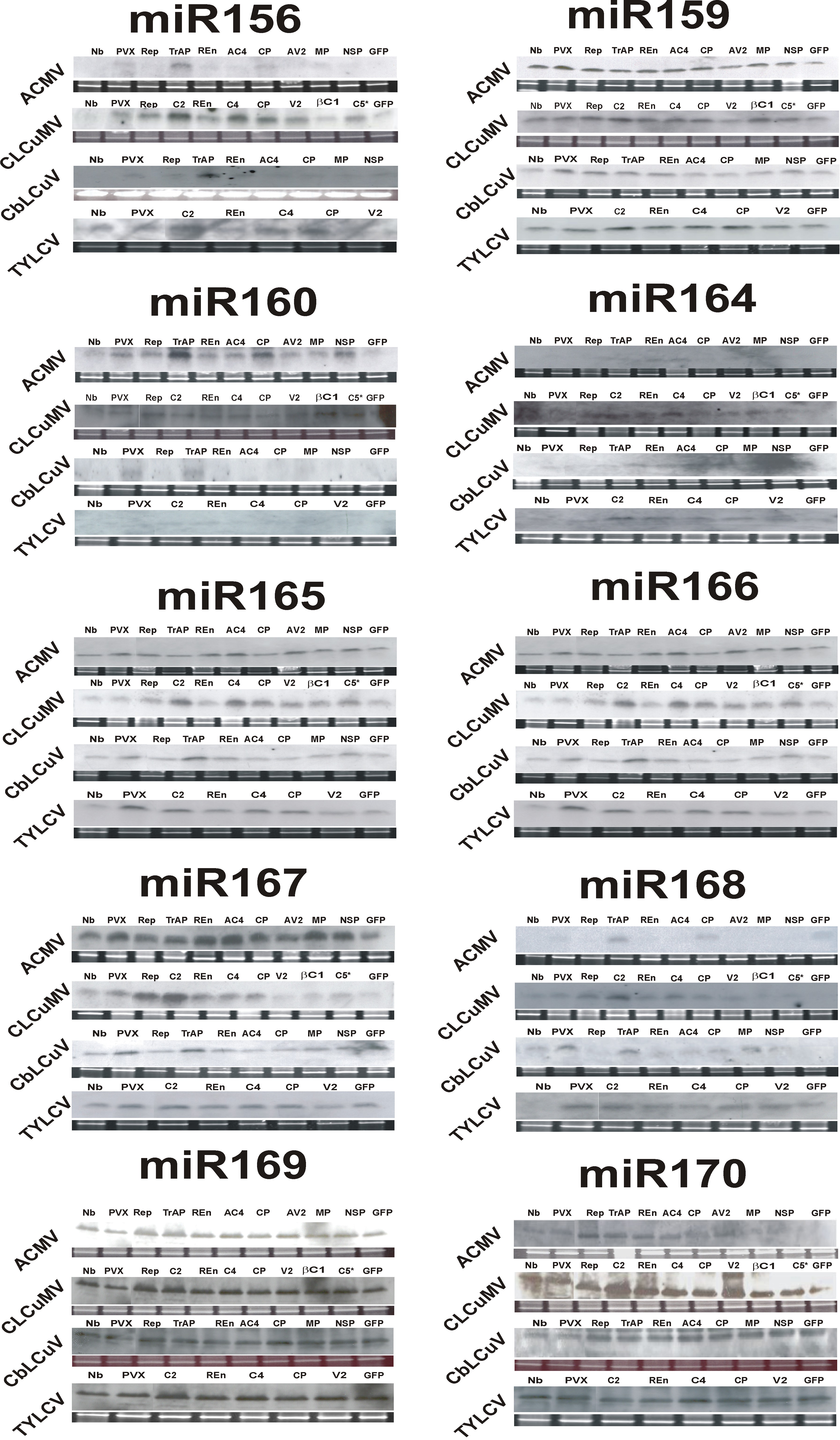

Supplement: Additional file 1 — Figure S1: Effects of PVX-mediated expression of begomovirus genes in N benthamiana on the levels of selected miRNAs. Northern blot analysis to detect the accumulation of selected miRNAs after infection with PVX and PVX expressing begomovirus-encoded genes. The genes used were those encoding the replication associated protein (Rep), the transcriptional activator protein (TrAP), the C2 protein (C2), the replication enhancer protein (REn), the (A)C4 protein [(A)C4], the coat protein (CP), the (A)V2 protein [(A)V2], the nuclear shuttle protein (NSP) and the movement protein (MP) Additionally the gene encoding the hypothetical protein C5 (C5*) of Cotton leaf curl Kokhran virus and the green fluorescence protein (GFP) were expressed from the PVX. Shown below the northern blots in each case are the rRNA bands of the ethidium bromide-stained agarose gels that were used to normalize the data for loading. [file 1743-422X-8-238-S1.JPEG]
