# Supplementary material for: Comparison of phenotypes produced in response to transient expression of genes encoded by four distinct begomoviruses in Nicotiana benthamiana and their correlation with the levels of developmental miRNAs
Source: Virol J. 2011 May 19;8:238. doi: 10.1186/1743-422X-8-238 (PMC3166278; doi:10.1186/1743-422X-8-238)
Supplement: Additional file 2 — Table S1: Primers used to PCR amplify begomovirus genes and gfpfor cloning in the PVX vector. [file 1743-422X-8-238-S2.DOC]

**Table S1 Primers used to amplify begomovirus genes and *gfp*** for cloning in PVX vector

| **Primer Name** | **Sequence** | **Virus/gene** |
| --- | --- | --- |
| P-AC-AV1-F  P-AC-AV1-R | CCATCGATATGTCGAAGCGACCAGGAGA  ACGCGTCGACTTAATTGCCAATACTGTCAT | ACMV/*av1* |
| P-AC-AV2-F  P-AC-AV2-R | CCATCGATATGTGGGATCCACTGGTGAA  ACGCGTCGA**C**CTATACATCCTGTACATCAT | ACMV/*av2* |
| P-AC-AC1-F  P-AC-AC1-R | TCCCCCGGGATGAGGACTCCTCGTTTTAG  ACGCGTCGACCTACGCCGGATGGCTCGCTTC | ACMV/*rep* |
| P-AC-AC2-F  P-AC-AC2-R | CCATCGATATGCAATCTTCATCACCCTC  ACGCGTCGACCTAAGACCCCTTAAGAAACG | ACMV/*trap* |
| P-AC-AC3-F  P-AC-AC3-R | CCATCGATATGGATTTACGCACAGGGGA  ACGCGTCGA**C**TTAATAAACATTGAATTGTA | ACMV/*ren* |
| P-AC-AC4-F  P-AC-AC4-R | TCCCCCGGGATGTCTTTCTCACATACCCA  ACGCGTCGA**C**CTAATTCCCTAATGACATTA | ACMV/*ac4* |
| P-AC-BC1-F  P-AC-BC1-R | CCATCGATATGGATACATCTGTACCTGT  TCCCCCGGGTCATAAATCTTTATGGCTAC | ACMV/*mp* |
| P-AC-BV1-F  P-AC-BV1-R | TCCCCCGGGATGTATAGTATCAGGAAGCA  ACGCGTCGA**C**TCATCCAACATAGGATGTTT | ACMV/*nsp* |
| PK3 V1 Cla  PK3 V1 R Sal | TTACATCGATATGTCGAAGCGAGCTGCCGA  TTCAGTCGACTCAATTCGTTACAGAGTCAT | CLCuMV/*cp* |
| PK3V2 F Cla  PK3V2 R Sal | AAATATCGATATGTGGGATCCACTATTAAAC  TCTGGTCGACCTATACATGGGCCTGTTTGT | CLCuMV/*v2* |
| PK3 C1 F Sma  PK3C1 R Sma | ACCACCCGGGATGGCTCCCCCCAAACGTTTT  ACACCCGGGTCAAGATCTACTCTCCTCCTCC | CLCuMV/*rep* |
| PK3 C2 F Sma  PK3 C2 R Sal | GCCTCCCGGGATGCAATCTTCATCACTCTC  TGCCGTCGACATGGATTCACGCACAGGGGA | CLCuMV/*trap* |
| PK3 C3 F Cla  PK3 C3 R Sal | TTACATCGATATGGATTCACGCACAGGGGA  TCTTGTCGACTAATGAAGTTTGAATTTTATTT | CLCuMV/*ren* |
| PK3 C4 R Sal  PK3 C4 F Sma | TCTTGTCGACCTAGTTCCTTAATGACTCT  TTACCCCGGGATGGGAGCCCTCATCTCCAT | CLCuMV/*c4* |
| β C1 Cla F  β C1 Sma R | ATAAATCGATATGACAACGAGCGGAACAAA  TGTTCCCGGGTTAAACGGTGAACTTTTTATT | CLCuMB/*βc1* |
| Cab BV1 Cla F  Cab BV1 Sal R | CTGATCGATATGTATCCTACAAAGTTTAG  TTATGTCGACTTAACCTAAATAATCAAGAT | CbLCuV/*nsp* |
| Cab BC1 Cla F  Cab BC1 Sal R | ATTTATCGATATGAATTCTCAGTTAGCGAAT  ACACGTCGACTTATTGCAATGACTTTGGTTG | CbLCuV/mp |
| Cab AC1 Cla F  Cab AC1 Sal R | ATACATCGATATGAAGCTCTTCAGATGCTT  TCTCGTCGACTTATAATTGCGAGAGCCTC | CbLCuV/*rep* |
| Cab AC4 Cla F  Cab AC4 Sal R | ATACATCGATATGAAGCTCTTCAGATGCTTC  TCTCGTCGACTTATAATTGCGAGAGCCTC | CbLCuV/*ac4* |
| Cab AC2 Cla F  Cab AC2 Sal R | ACTAATCGATATGCAAAATTCATCACTCTTG  GCATGTCGACCTACTTAAATATGTCGGCCCA | CbLCuV/*trap* |
| Cab AC3 Cla F  Cab AC3 Sal R | TGCGATCGATATGGATTCACGCACCGGGGA  CGATGTCGACTTAATAAATTTTGAAATTTAT | CbLCuV/*ren* |
| Cab AV1 Cla F  Cab AV1 Sal R | ATTTATCGATATGCTTTACTTCGAAATGCC  TCAAGTCGACTTAATTTGTTATCGAATCGT | CbLCuV /*cp* |
| TYLC V2ClaF  TYLC V2 SalR | GTCTATCGATATGTGGGATCCACTTCTAAAT  ACGGGTCGACTCAGTGCTTCGATACATTCTG | TYLCV/*v2* |
| TYLCV1SmaF  TYLCV1 SalR | GCCCCCCGGGATGTCGAAGCGACCAGGCGA  ATAAGTCGACTTAATTTGATATTGAATCATA | TYLCV/*cp* |
| TYLC C3 ClaF  TYLC C3 SalF | TGCAATCGATATGGATTCACGCACAGGGG  CAATGTCGACTTAATAAAATTTATATTTTAT | TYLCV/*ren* |
| TYLC C2 ClaF  TYLC C2 SalR | GCTCATCGATATGCAACCTTCGTCACCCTCT  AACTGTCGACCTAAATACTTTTAAGAAACG | TYLCV/*trap* |
| TYLC C1 ClaF  TYLCC1SmaR | CATTATCGATATGGCTCCCCCTAAGCGCTT  GTCTCCCGGGTTACGCCTTATTGGTTTCT | TYLCV/*rep* |
| PCLC5 F Cla  PCLC5 R Sal | ACCTATCGAT ATGAACATTCTTCACAGTCGC  GTAAGTCGACCTAGGAATTATGTCGAAGC | CLCuKoV/*c5* |
| TYLC C4 ClaF  TYLC4 SmaR | CTCCATCGATATGGGGAACCACATCTCCAT  TCTTCCCGGGTTAATTCTTTAATGATTCTAAG | TYLCV/*c4* |
| P GFP Cla F  P GFP Sal R | AGATATCGATATGAGTAAAGGAGAAGAAC  GGGTCGACTTATTTGTATAGTTCATCCATG | *gfp* |
